# Supplementary material for: Complex plumages spur rapid color diversification in kingfishers (Aves: Alcedinidae)
Source: eLife. 2023 Apr 21;12:e83426. doi: 10.7554/eLife.83426 (PMC10121218; doi:10.7554/eLife.83426)
Supplement: Supplementary file 1. — (a) Evolutionary model fits using multivariate color data sets. (b) Predictors of rates of color evolution when analyzing males and females separately. (c) Evolutionary rates tests using discrete plumage complexity scores. (d) Predictors of plumage complexity for males and females analyzed separately. (e) Testing predictors of shifts in average plumage hue and brightness. (f) Full PGLS regression results for predictors of chromatic rate variation. (g) Full PGLS regression results for predictors of achromatic rate variation. [file elife-83426-supp1.docx]

# Supporting Information for:

**Complex plumages spur rapid color diversification in kingfishers (Aves: Alcedinidae)**

Authors: Chad M. Eliason^1,2^, Jenna M. McCullough^3^, Shannon J. Hackett^2^, Michael J. Andersen^3^

^1^Grainger Bioinformatics Center, Field Museum of Natural History, Chicago, IL USA

^2^Negaunee Integrative Research Center, Field Museum of Natural History, Chicago, IL USA

^3^Department of Biology and Museum of Southwestern Biology, University of New Mexico, Albuquerque, NM USA

## Supplementary Tables

**Supplementary file 1a. Evolutionary model fits using multivariate color data sets.** Models compared were a Brownian motion (BM) model and Pagel's λ model. Models were fit separately for each sex, as well as using chromatic and achromatic variables (i.e., luminance). Generalized information criteria (GIC) scores were calculated using function in the RPANDA R package.

| **Sex** | **Data type** | **GIC BM** | **GIC λ** | **GIC wt.** | **Pagel's λ** |
| --- | --- | --- | --- | --- | --- |
| Both | Chromatic | 13253 | 12027 | 1.00 | 0.24 |
|  | Achromatic | 9295 | 8844 | 1.00 | 0.37 |
| Male | Chromatic | 12881 | 11636 | 1.00 | 0.21 |
|  | Achromatic | 8488 | 8079 | 1.00 | 0.33 |
| Female | Chromatic | 14028 | 12585 | 1.00 | 0.15 |
|  | Achromatic | 9096 | 8586 | 1.00 | 0.29 |

**Supplementary file 1b. Predictors of rates of color evolution when analyzing males and females separately.** Models were fit using PGLS in the phylolm R package, with species-specific evolutionary rates as the response variable and complexity metrics (c1, c2, c3), island-dwelling, natural log body mass, and number of sympatric species as predictors. The best-fitting models were determined using a stepwise AIC-based procedure (i.e., using the phylostep function). Significant predictors in the most parsimonious models are indicated in bold.

| **Sex** | **Response** | **Predictor** | **β** | **P** | **R^2^** | **Signal** |
| --- | --- | --- | --- | --- | --- | --- |
| Male | **Chromatic rate** | **Color volume (c2)** | **0.27 ± 0.13** | **0.03** | 0.46 | 0.00 |
|  |  | **# unique patches (c3)** | **0.46 ± 0.13** | **<0.01** |  |  |
|  | **Achromatic rate** | **Interpatch dist. (c1)** | **0.37 ± 0.13** | **<0.01** | 0.17 | 0.00 |
|  |  | # unique patches (c3) | -0.18 ± 0.13 | 0.15 |  |  |
|  |  | ln mass | 0.21 ± 0.19 | 0.09 |  |  |
| Female | **Chromatic rate** | **Interpatch dist. (c1)** | **0.46 ± 0.16** | **<0.01** | 0.59 | 0.00 |
|  |  | Color volume (c2) | 0.29 ± 0.16 | 0.08 |  |  |
|  |  | **Insularity** | **0.49 ± 0.17** | **<0.01** |  |  |
|  | **Achromatic rate** | **Lightness range (c2)** | **0.37 ± 0.11** | **<0.01** | 0.14 | 0.02 |

**Supplementary file 1c. Evolutionary rates tests using discrete plumage complexity scores.** Results of fitting rate ratio tests in compare.evol.rates using binary complexity scores (i.e., low versus high) obtained with kmeans clustering. Significant tests (P < 0.05) indicated in bold.

| **Sex** | **Data type** | **Complexity metric** | $\boldsymbol{\sigma}_{\text{simple}}^{\boldsymbol{2}}$ | $\boldsymbol{\sigma}_{\text{complex}}^{\boldsymbol{2}}$ | **P** | **N** |
| --- | --- | --- | --- | --- | --- | --- |
| Both | Chromatic | **Mean interpatch distance (c1)** | **0.13** | **0.21** | **0.04** | **72** |
|  |  | **Color volume (c2)** | **0.11** | **0.29** | **<0.01** | **72** |
|  |  | **# unique patches (c3)** | **0.09** | **0.22** | **<0.01** | **72** |
|  | Achromatic | Mean interpatch distance (c1) | 0.76 | 1.06 | 0.11 | 72 |
|  |  | **Lightness range (c2)** | **0.67** | **1.05** | **0.03** | **72** |
|  |  | # unique patches (c3) | 1.09 | 0.79 | 0.22 | 72 |
| Male | Chromatic | **Mean interpatch distance (c1)** | **0.13** | **0.21** | **0.02** | **63** |
|  |  | **Color volume (c2)** | **0.15** | **0.35** | **0.01** | **63** |
|  |  | **# unique patches (c3)** | **0.10** | **0.22** | **<0.01** | **63** |
|  | Achromatic | Mean interpatch distance (c1) | 0.91 | 1.17 | 0.23 | 63 |
|  |  | Lightness range (c2) | 0.82 | 1.16 | 0.1 | 63 |
|  |  | # unique patches (c3) | 1.00 | 1.04 | 0.87 | 63 |
| Female | Chromatic | **Mean interpatch distance (c1)** | **0.13** | **0.24** | **<0.01** | **68** |
|  |  | **Color volume (c2)** | **0.14** | **0.44** | **<0.01** | **68** |
|  |  | **# unique patches (c3)** | **0.06** | **0.21** | **<0.01** | **68** |
|  | Achromatic | Mean interpatch distance (c1) | 0.75 | 1.10 | 0.05 | 68 |
|  |  | **Lightness range (c2)** | **0.67** | **1.03** | **0.02** | **68** |
|  |  | # unique patches (c3) | 0.83 | 0.91 | 0.61 | 68 |

**Supplementary file 1d. Predictors of plumage complexity for males and females analyzed separately.** Models were fit for both chromatic (i.e., hue and saturation) and achromatic variables (i.e., lightness) using PGLS in the phylolm R package. Different complexity metrics were set as the response variable, and island-dwelling, body mass (ln mass), and number of sympatric species were used as predictors. The best-fitting models were determined using a stepwise AIC-based procedure (i.e., using the phylostep function). Significant predictors are indicated in bold. See Table 2 for sex-specific results.

| **Sex** | **Data type** | **Response** | **Predictor** | **Effect** | **P** |
| --- | --- | --- | --- | --- | --- |
| Male | Chromatic | Interpatch dist.(c1) | ln mass | -0.29 ± 0.15 | 0.06 |
|  |  | **# unique patches (c3)** | **ln mass** | **-0.31 ± 0.15** | **0.05** |
|  | Achromatic | Lightness range (c2) | # symp. species | -0.23 ± 0.12 | 0.08 |
| Female | Chromatic | **Interpatch dist. (c1)** | **ln mass** | **-0.38 ± 0.14** | **<0.01** |
|  |  | **Color volume (c2)** | **ln mass** | **-0.36 ± 0.14** | **0.01** |
|  |  | **# unique patches (c3)** | **ln mass** | **-0.33 ± 0.15** | **0.04** |
|  | Achromatic | Interpatch dist. (c1) | islands1 | -0.41 ± 0.28 | 0.15 |
|  |  | Interpatch dist. (c1) | # symp. species | -0.18 ± 0.13 | 0.17 |
|  |  | **Interpatch dist. (c1)** | **ln mass** | **-0.28 ± 0.12** | **0.02** |
|  |  | **Lightness range (c2)** | **Insularity** | **-0.61 ± 0.27** | **0.03** |
|  |  | **Lightness range (c2)** | **# symp. species** | **-0.36 ± 0.13** | **<0.01** |
|  |  | # unique patches (c3) | ln mass | -0.27 ± 0.18 | 0.14 |

**Supplementary file 1e. Testing predictors of shifts in average plumage hue and brightness.** Results of multivariate distance-based d-PGLS tests testing for convergence in overall plumage coloration on islands. P values were calculated with a permutation approach using 999 iterations. Significant predictors shown in bold.

| **Sex** | **Response** | **Predictor** | **F** | **P** | **N_traits_** | **N_species_** |
| --- | --- | --- | --- | --- | --- | --- |
| Male | Multivariate color | **Islands** | **2.44** | **0.03** | **66** | **63** |
|  |  | # sympatric species | 0.72 | 0.61 | 66 | 63 |
|  |  | ln body mass | 1.10 | 0.30 | 66 | 63 |
|  | Multivariate lightness | Islands | 1.18 | 0.26 | 22 | 63 |
|  |  | # sympatric species | 0.97 | 0.41 | 22 | 63 |
|  |  | ln body mass | 1.08 | 0.34 | 22 | 63 |
| Female | Multivariate color | Islands | 1.18 | 0.27 | 66 | 68 |
|  |  | # sympatric species | 0.58 | 0.73 | 66 | 68 |
|  |  | ln body mass | 1.58 | 0.15 | 66 | 68 |
|  | Multivariate lightness | Islands | 0.95 | 0.44 | 22 | 68 |
|  |  | # sympatric species | 1.76 | 0.08 | 22 | 68 |
|  |  | ln body mass | 1.79 | 0.07 | 22 | 68 |

**Supplementary file 1f. Full PGLS regression results for predictors of chromatic rate variation.** Models were fit using PGLS in the phylolm R package, with species-specific evolutionary rates as the response variable and complexity metrics (c1, c2, c3), island-dwelling, natural log body mass, and number of sympatric species as predictors. Estimates ± 1 std. error are shown in columns 2-7. Cells for predictors not in that specific model are left blank. Models are sorted by increasing AIC scores, with the best models at the top of the list.

| **model** | **Intercept** | **c1** | **c2** | **c3** | **insularity** | **# symp. spp.** | **ln mass** | **AIC** | **ΔAIC** | **λ** | **σ^2^** |
| --- | --- | --- | --- | --- | --- | --- | --- | --- | --- | --- | --- |
| 26 | -0.14 ± 0.11 | 0.41 ± 0.13 |  | 0.24 ± 0.2 | 0.49 ± 0.11 |  |  | 171.97 | 0 | 0.00 | 0.01 |
| 42 | -0.14 ± 0.11 | 0.27 ± 0.17 | 0.18 ± 0.15 | 0.23 ± 0.13 | 0.48 ± 0.2 |  |  | 172.52 | 0.55 | 0.00 | 0.01 |
| 49 | -0.13 ± 0.11 | 0.35 ± 0.14 |  | 0.23 ± 0.21 | 0.46 ± 0.11 |  | -0.11 ± 0.14 | 172.9 | 0.93 | 0.00 | 0.01 |
| 32 | -0.13 ± 0.11 |  | 0.34 ± 0.12 | 0.33 ± 0.2 | 0.44 ± 0.11 |  |  | 173.16 | 1.19 | 0.00 | 0.01 |
| 48 | -0.12 ± 0.11 | 0.4 ± 0.13 |  | 0.26 ± 0.23 | 0.41 ± 0.1 | -0.08 ± 0.11 |  | 173.25 | 1.28 | 0.00 | 0.01 |
| 53 | -0.12 ± 0.11 |  | 0.29 ± 0.12 | 0.3 ± 0.2 | 0.42 ± 0.11 |  | -0.14 ± 0.12 | 173.35 | 1.38 | 0.00 | 0.01 |
| 9 | -0.17 ± 0.11 | 0.57 ± 0.09 |  |  | 0.59 ± 0.09 |  |  | 173.48 | 1.51 | 0.00 | 0.02 |
| 58 | -0.13 ± 0.11 | 0.23 ± 0.18 | 0.17 ± 0.15 | 0.22 ± 0.13 | 0.46 ± 0.21 |  | -0.1 ± 0.11 | 173.6 | 1.63 | 0.00 | 0.01 |
| 23 | -0.17 ± 0.11 | 0.42 ± 0.15 | 0.19 ± 0.15 |  | 0.58 ± 0.11 |  |  | 173.84 | 1.87 | 0.00 | 0.01 |
| 57 | -0.12 ± 0.11 | 0.27 ± 0.17 | 0.17 ± 0.15 | 0.25 ± 0.13 | 0.41 ± 0.23 | -0.08 ± 0.1 |  | 173.87 | 1.9 | 0.00 | 0.01 |
| 30 | -0.16 ± 0.11 | 0.5 ± 0.11 |  |  | 0.55 ± 0.11 |  | -0.13 ± 0.2 | 174.14 | 2.17 | 0.00 | 0.01 |
| 61 | -0.11 ± 0.11 | 0.34 ± 0.14 |  | 0.25 ± 0.23 | 0.38 ± 0.1 | -0.08 ± 0.11 | -0.11 ± 0.11 | 174.18 | 2.21 | 0.00 | 0.01 |
| 52 | -0.11 ± 0.11 |  | 0.33 ± 0.12 | 0.35 ± 0.23 | 0.36 ± 0.1 | -0.08 ± 0.11 |  | 174.44 | 2.47 | 0.00 | 0.01 |
| 27 | 0 ± 0.09 | 0.36 ± 0.13 |  | 0.34 ± 0.09 |  | -0.16 ± 0.13 |  | 174.63 | 2.66 | 0.00 | 0.01 |
| 62 | -0.1 ± 0.11 |  | 0.28 ± 0.12 | 0.32 ± 0.23 | 0.34 ± 0.1 | -0.08 ± 0.11 | -0.14 ± 0.11 | 174.64 | 2.67 | 0.00 | 0.01 |
| 46 | -0.16 ± 0.11 | 0.36 ± 0.16 | 0.18 ± 0.15 |  | 0.55 ± 0.11 |  | -0.12 ± 0.16 | 174.69 | 2.72 | 0.00 | 0.01 |
| 63 | -0.11 ± 0.11 | 0.22 ± 0.18 | 0.16 ± 0.15 | 0.24 ± 0.13 | 0.38 ± 0.23 | -0.08 ± 0.1 | -0.1 ± 0.11 | 174.94 | 2.97 | 0.00 | 0.01 |
| 54 | 0 ± 0.09 |  | 0.26 ± 0.12 | 0.37 ± 0.09 |  | -0.15 ± 0.09 | -0.15 ± 0.12 | 175.04 | 3.07 | 0.00 | 0.01 |
| 33 | 0 ± 0.09 |  | 0.31 ± 0.11 | 0.41 ± 0.09 |  | -0.15 ± 0.11 |  | 175.13 | 3.16 | 0.00 | 0.02 |
| 50 | 0 ± 0.09 | 0.3 ± 0.14 |  | 0.32 ± 0.09 |  | -0.15 ± 0.09 | -0.13 ± 0.14 | 175.15 | 3.18 | 0.00 | 0.01 |
| 43 | 0 ± 0.09 | 0.23 ± 0.17 | 0.17 ± 0.15 | 0.33 ± 0.13 |  | -0.16 ± 0.09 |  | 175.29 | 3.32 | 0.00 | 0.01 |
| 29 | -0.16 ± 0.11 | 0.58 ± 0.09 |  |  | 0.55 ± 0.11 | -0.04 ± 0.09 |  | 175.3 | 3.33 | 0.00 | 0.02 |
| 45 | -0.16 ± 0.11 | 0.42 ± 0.16 | 0.19 ± 0.16 |  | 0.55 ± 0.1 | -0.04 ± 0.11 |  | 175.68 | 3.71 | 0.00 | 0.01 |
| 34 | 0 ± 0.09 |  | 0.26 ± 0.12 | 0.35 ± 0.11 |  |  | -0.16 ± 0.12 | 175.73 | 3.76 | 0.00 | 0.02 |
| 8 | 0 ± 0.09 | 0.37 ± 0.13 |  | 0.33 ± 0.09 |  |  |  | 175.81 | 3.84 | 0.00 | 0.02 |
| 51 | -0.15 ± 0.11 | 0.51 ± 0.11 |  |  | 0.52 ± 0.11 | -0.04 ± 0.11 | -0.13 ± 0.11 | 175.95 | 3.98 | 0.00 | 0.01 |
| 59 | 0 ± 0.09 | 0.18 ± 0.18 | 0.16 ± 0.16 | 0.32 ± 0.13 |  | -0.15 ± 0.11 | -0.12 ± 0.09 | 175.98 | 4.01 | 0.00 | 0.01 |
| 12 | 0 ± 0.09 |  | 0.32 ± 0.12 | 0.39 ± 0.09 |  |  |  | 175.99 | 4.02 | 0.00 | 0.02 |
| 28 | 0 ± 0.09 | 0.3 ± 0.14 |  | 0.31 ± 0.11 |  |  | -0.14 ± 0.13 | 176.11 | 4.14 | 0.00 | 0.02 |
| 22 | 0 ± 0.09 | 0.22 ± 0.18 | 0.19 ± 0.16 | 0.31 ± 0.13 |  |  |  | 176.34 | 4.37 | 0.00 | 0.02 |
| 60 | -0.15 ± 0.11 | 0.37 ± 0.16 | 0.18 ± 0.16 |  | 0.51 ± 0.1 | -0.04 ± 0.11 | -0.12 ± 0.11 | 176.52 | 4.55 | 0.00 | 0.01 |
| 44 | 0 ± 0.09 | 0.17 ± 0.18 | 0.17 ± 0.16 | 0.3 ± 0.13 |  |  | -0.14 ± 0.18 | 176.83 | 4.86 | 0.00 | 0.01 |
| 39 | -0.11 ± 0.11 |  |  | 0.43 ± 0.11 | 0.37 ± 0.11 |  | -0.23 ± 0.21 | 177.49 | 5.52 | 0.00 | 0.02 |
| 36 | -0.15 ± 0.11 |  | 0.43 ± 0.21 |  | 0.53 ± 0.11 |  | -0.2 ± 0.21 | 177.8 | 5.83 | 0.00 | 0.02 |
| 40 | 0 ± 0.09 |  |  | 0.49 ± 0.11 |  | -0.15 ± 0.11 | -0.23 ± 0.09 | 177.96 | 5.99 | 0.00 | 0.02 |
| 13 | -0.22 ± 0.25 |  | 0.43 ± 0.21 |  | 0.49 ± 0.11 |  |  | 178.1 | 6.13 | 0.33 | 0.02 |
| 56 | -0.08 ± 0.11 |  |  | 0.46 ± 0.1 | 0.27 ± 0.11 | -0.1 ± 0.11 | -0.22 ± 0.11 | 178.51 | 6.54 | 0.00 | 0.02 |
| 18 | 0 ± 0.09 |  |  | 0.47 ± 0.09 |  |  | -0.24 ± 0.09 | 178.64 | 6.67 | 0.00 | 0.02 |
| 31 | 0 ± 0.09 | 0.5 ± 0.12 |  |  |  | -0.14 ± 0.12 | -0.17 ± 0.09 | 179.6 | 7.63 | 0.00 | 0.02 |
| 35 | -0.22 ± 0.27 |  | 0.43 ± 0.25 |  | 0.41 ± 0.27 | -0.07 ± 0.11 |  | 179.63 | 7.66 | 0.36 | 0.02 |
| 11 | 0 ± 0.09 | 0.49 ± 0.12 |  |  |  |  | -0.18 ± 0.09 | 179.72 | 7.75 | 0.00 | 0.02 |
| 55 | -0.15 ± 0.11 |  | 0.43 ± 0.23 |  | 0.51 ± 0.11 | -0.02 ± 0.11 | -0.2 ± 0.11 | 179.74 | 7.77 | 0.00 | 0.02 |
| 10 | 0 ± 0.09 | 0.6 ± 0.1 |  |  |  | -0.14 ± 0.1 |  | 179.77 | 7.8 | 0.00 | 0.02 |
| 16 | -0.11 ± 0.14 |  |  | 0.49 ± 0.14 | 0.38 ± 0.1 |  |  | 179.78 | 7.81 | 0.05 | 0.02 |
| 17 | -0.02 ± 0.15 |  |  | 0.52 ± 0.15 |  | -0.16 ± 0.1 |  | 180.04 | 8.07 | 0.09 | 0.02 |
| 1 | 0 ± 0.1 | 0.59 ± 0.1 |  |  |  |  |  | 180.06 | 8.09 | 0.00 | 0.02 |
| 24 | 0 ± 0.09 | 0.44 ± 0.16 | 0.2 ± 0.16 |  |  | -0.14 ± 0.16 |  | 180.12 | 8.15 | 0.00 | 0.02 |
| 47 | 0 ± 0.09 | 0.36 ± 0.17 | 0.18 ± 0.16 |  |  | -0.13 ± 0.09 | -0.16 ± 0.17 | 180.19 | 8.22 | 0.00 | 0.02 |
| 25 | 0 ± 0.09 | 0.34 ± 0.17 | 0.19 ± 0.16 |  |  |  | -0.16 ± 0.16 | 180.21 | 8.24 | 0.00 | 0.02 |
| 7 | 0 ± 0.1 | 0.42 ± 0.16 | 0.21 ± 0.16 |  |  |  |  | 180.3 | 8.33 | 0.00 | 0.02 |
| 14 | -0.14 ± 0.29 |  | 0.41 ± 0.09 |  |  | -0.16 ± 0.09 |  | 180.4 | 8.43 | 0.44 | 0.02 |
| 38 | -0.09 ± 0.15 |  |  | 0.51 ± 0.11 | 0.27 ± 0.15 | -0.11 ± 0.11 |  | 180.74 | 8.77 | 0.05 | 0.02 |
| 3 | -0.01 ± 0.14 |  |  | 0.51 ± 0.11 |  |  |  | 180.95 | 8.98 | 0.07 | 0.02 |
| 2 | -0.12 ± 0.27 |  | 0.41 ± 0.27 |  |  |  |  | 181.52 | 9.55 | 0.37 | 0.02 |
| 37 | -0.12 ± 0.24 |  | 0.39 ± 0.09 |  |  | -0.16 ± 0.11 | -0.15 ± 0.09 | 181.56 | 9.59 | 0.30 | 0.02 |
| 15 | 0 ± 0.1 |  | 0.43 ± 0.11 |  |  |  | -0.24 ± 0.1 | 182.36 | 10.39 | 0.00 | 0.02 |
| 20 | -0.21 ± 0.22 |  |  |  | 0.45 ± 0.23 |  | -0.3 ± 0.22 | 190.06 | 18.09 | 0.21 | 0.02 |
| 4 | -0.25 ± 0.33 |  |  |  | 0.41 ± 0.33 |  |  | 190.96 | 18.99 | 0.45 | 0.03 |
| 5 | -0.18 ± 0.35 |  |  |  |  | -0.16 ± 0.1 |  | 191.44 | 19.47 | 0.51 | 0.03 |
| 21 | -0.14 ± 0.27 |  |  |  |  | -0.15 ± 0.15 | -0.25 ± 0.27 | 191.55 | 19.58 | 0.34 | 0.02 |
| 41 | -0.2 ± 0.24 |  |  |  | 0.37 ± 0.24 | -0.07 ± 0.26 | -0.28 ± 0.11 | 191.69 | 19.72 | 0.25 | 0.02 |
| 6 | -0.11 ± 0.25 |  |  |  |  |  | -0.28 ± 0.25 | 191.88 | 19.91 | 0.27 | 0.02 |
| 19 | -0.24 ± 0.34 |  |  |  | 0.29 ± 0.27 | -0.1 ± 0.12 |  | 192.26 | 20.29 | 0.47 | 0.03 |

**Supplementary file 1g. Full PGLS regression results for predictors of achromatic rate variation.** Models were fit using PGLS in the phylolm R package, with species-specific evolutionary rates as the response variable and complexity metrics (c1, c2, c3), island-dwelling, natural log body mass, and number of sympatric species as predictors. Estimates ± 1 std. error are shown in columns 2-7. Cells for predictors not in that specific model are left blank. Models are sorted by increasing AIC scores, with the best models at the top of the list.

| **model** | **Intercept** | **c1** | **c2** | **c3** | **insularity** | **# symp. spp.** | **ln mass** | **AIC** | **ΔAIC** | **λ** | **σ^2^** |
| --- | --- | --- | --- | --- | --- | --- | --- | --- | --- | --- | --- |
| 15 | 0 ± 0.11 |  | 0.31 ± 0.11 |  |  |  | 0.23 ± 0.11 | 202.49 | 0 | 0.00 | 0.02 |
| 34 | 0 ± 0.11 |  | 0.35 ± 0.11 | -0.16 ± 0.11 |  |  | 0.22 ± 0.11 | 202.51 | 0.02 | 0.00 | 0.02 |
| 12 | -0.06 ± 0.19 |  | 0.35 ± 0.11 | -0.18 ± 0.19 |  |  |  | 202.71 | 0.22 | 0.11 | 0.02 |
| 28 | 0 ± 0.11 | 0.35 ± 0.12 |  | -0.18 ± 0.11 |  |  | 0.24 ± 0.12 | 203.22 | 0.73 | 0.00 | 0.02 |
| 2 | -0.04 ± 0.17 |  | 0.31 ± 0.17 |  |  |  |  | 203.28 | 0.79 | 0.08 | 0.02 |
| 44 | 0 ± 0.11 | 0.18 ± 0.17 | 0.22 ± 0.17 | -0.18 ± 0.12 |  |  | 0.23 ± 0.17 | 203.42 | 0.93 | 0.00 | 0.02 |
| 11 | 0 ± 0.11 | 0.29 ± 0.11 |  |  |  |  | 0.25 ± 0.11 | 203.73 | 1.24 | 0.00 | 0.02 |
| 36 | -0.06 ± 0.13 |  | 0.33 ± 0.25 |  | 0.2 ± 0.13 |  | 0.24 ± 0.25 | 203.84 | 1.35 | 0.00 | 0.02 |
| 25 | 0 ± 0.11 | 0.12 ± 0.17 | 0.22 ± 0.17 |  |  |  | 0.24 ± 0.17 | 203.96 | 1.47 | 0.00 | 0.02 |
| 54 | 0 ± 0.11 |  | 0.37 ± 0.12 | -0.17 ± 0.11 |  | 0.07 ± 0.11 | 0.22 ± 0.12 | 204.08 | 1.59 | 0.00 | 0.02 |
| 53 | -0.04 ± 0.13 |  | 0.36 ± 0.12 | -0.15 ± 0.25 | 0.15 ± 0.11 |  | 0.23 ± 0.12 | 204.13 | 1.64 | 0.00 | 0.02 |
| 37 | 0 ± 0.11 |  | 0.33 ± 0.11 |  |  | 0.05 ± 0.12 | 0.23 ± 0.11 | 204.26 | 1.77 | 0.00 | 0.02 |
| 22 | -0.05 ± 0.18 | 0.1 ± 0.17 | 0.28 ± 0.17 | -0.19 ± 0.12 |  |  |  | 204.38 | 1.89 | 0.09 | 0.02 |
| 32 | -0.1 ± 0.2 |  | 0.36 ± 0.12 | -0.18 ± 0.26 | 0.12 ± 0.2 |  |  | 204.47 | 1.98 | 0.12 | 0.02 |
| 33 | -0.05 ± 0.18 |  | 0.36 ± 0.12 | -0.19 ± 0.11 |  | 0.05 ± 0.12 |  | 204.55 | 2.06 | 0.10 | 0.02 |
| 13 | -0.09 ± 0.19 |  | 0.32 ± 0.25 |  | 0.17 ± 0.11 |  |  | 204.81 | 2.32 | 0.09 | 0.02 |
| 55 | -0.09 ± 0.14 |  | 0.36 ± 0.28 |  | 0.31 ± 0.11 | 0.12 ± 0.14 | 0.25 ± 0.12 | 204.94 | 2.45 | 0.00 | 0.02 |
| 8 | -0.03 ± 0.16 | 0.31 ± 0.12 |  | -0.19 ± 0.16 |  |  |  | 204.99 | 2.5 | 0.06 | 0.02 |
| 49 | -0.03 ± 0.13 | 0.35 ± 0.12 |  | -0.17 ± 0.25 | 0.12 ± 0.12 |  | 0.25 ± 0.12 | 205 | 2.51 | 0.00 | 0.02 |
| 58 | -0.04 ± 0.13 | 0.18 ± 0.18 | 0.23 ± 0.17 | -0.17 ± 0.12 | 0.15 ± 0.25 |  | 0.25 ± 0.13 | 205.04 | 2.55 | 0.00 | 0.02 |
| 62 | -0.08 ± 0.14 |  | 0.39 ± 0.12 | -0.15 ± 0.28 | 0.27 ± 0.13 | 0.13 ± 0.11 | 0.24 ± 0.14 | 205.04 | 2.55 | 0.00 | 0.02 |
| 59 | 0 ± 0.11 | 0.16 ± 0.18 | 0.24 ± 0.18 | -0.18 ± 0.12 |  | 0.05 ± 0.11 | 0.23 ± 0.11 | 205.19 | 2.7 | 0.00 | 0.02 |
| 14 | -0.04 ± 0.17 |  | 0.31 ± 0.12 |  |  | 0.03 ± 0.12 |  | 205.2 | 2.71 | 0.07 | 0.02 |
| 7 | -0.04 ± 0.17 | 0.05 ± 0.17 | 0.27 ± 0.17 |  |  |  |  | 205.21 | 2.72 | 0.07 | 0.02 |
| 50 | 0 ± 0.11 | 0.35 ± 0.12 |  | -0.18 ± 0.11 |  | 0.01 ± 0.11 | 0.24 ± 0.12 | 205.22 | 2.73 | 0.00 | 0.02 |
| 30 | -0.05 ± 0.13 | 0.3 ± 0.12 |  |  | 0.17 ± 0.13 |  | 0.26 ± 0.25 | 205.26 | 2.77 | 0.00 | 0.02 |
| 46 | -0.06 ± 0.13 | 0.13 ± 0.17 | 0.24 ± 0.17 |  | 0.2 ± 0.12 |  | 0.26 ± 0.17 | 205.28 | 2.79 | 0.00 | 0.02 |
| 1 | -0.02 ± 0.16 | 0.25 ± 0.11 |  |  |  |  |  | 205.66 | 3.17 | 0.05 | 0.02 |
| 31 | 0 ± 0.11 | 0.29 ± 0.11 |  |  |  | -0.01 ± 0.11 | 0.25 ± 0.11 | 205.73 | 3.24 | 0.00 | 0.02 |
| 47 | 0 ± 0.11 | 0.11 ± 0.18 | 0.24 ± 0.18 |  |  | 0.04 ± 0.11 | 0.24 ± 0.18 | 205.85 | 3.36 | 0.00 | 0.02 |
| 52 | -0.11 ± 0.2 |  | 0.38 ± 0.12 | -0.18 ± 0.29 | 0.21 ± 0.13 | 0.09 ± 0.2 |  | 205.97 | 3.48 | 0.11 | 0.02 |
| 42 | -0.09 ± 0.2 | 0.09 ± 0.17 | 0.28 ± 0.17 | -0.19 ± 0.12 | 0.12 ± 0.26 |  |  | 206.17 | 3.68 | 0.11 | 0.02 |
| 43 | -0.05 ± 0.18 | 0.09 ± 0.18 | 0.29 ± 0.18 | -0.2 ± 0.12 |  | 0.04 ± 0.18 |  | 206.28 | 3.79 | 0.09 | 0.02 |
| 63 | -0.07 ± 0.14 | 0.14 ± 0.18 | 0.28 ± 0.18 | -0.17 ± 0.12 | 0.25 ± 0.28 | 0.11 ± 0.13 | 0.25 ± 0.12 | 206.33 | 3.84 | 0.00 | 0.02 |
| 35 | -0.11 ± 0.19 |  | 0.34 ± 0.29 |  | 0.26 ± 0.19 | 0.09 ± 0.12 |  | 206.35 | 3.86 | 0.09 | 0.02 |
| 60 | -0.09 ± 0.14 | 0.09 ± 0.18 | 0.29 ± 0.19 |  | 0.3 ± 0.13 | 0.1 ± 0.12 | 0.26 ± 0.14 | 206.64 | 4.15 | 0.00 | 0.02 |
| 23 | -0.09 ± 0.19 | 0.05 ± 0.17 | 0.28 ± 0.17 |  | 0.17 ± 0.19 |  |  | 206.74 | 4.25 | 0.09 | 0.02 |
| 26 | -0.05 ± 0.18 | 0.31 ± 0.12 |  | -0.19 ± 0.26 | 0.07 ± 0.18 |  |  | 206.91 | 4.42 | 0.07 | 0.02 |
| 61 | -0.04 ± 0.14 | 0.35 ± 0.12 |  | -0.17 ± 0.28 | 0.14 ± 0.12 | 0.03 ± 0.12 | 0.26 ± 0.14 | 206.93 | 4.44 | 0.00 | 0.02 |
| 27 | -0.03 ± 0.16 | 0.31 ± 0.12 |  | -0.19 ± 0.11 |  | -0.01 ± 0.12 |  | 206.98 | 4.49 | 0.06 | 0.02 |
| 24 | -0.04 ± 0.17 | 0.04 ± 0.18 | 0.28 ± 0.18 |  |  | 0.03 ± 0.18 |  | 207.15 | 4.66 | 0.07 | 0.02 |
| 51 | -0.06 ± 0.14 | 0.3 ± 0.12 |  |  | 0.19 ± 0.12 | 0.03 ± 0.14 | 0.27 ± 0.12 | 207.21 | 4.72 | 0.00 | 0.02 |
| 9 | -0.06 ± 0.18 | 0.26 ± 0.11 |  |  | 0.13 ± 0.11 |  |  | 207.42 | 4.93 | 0.06 | 0.02 |
| 10 | -0.03 ± 0.16 | 0.25 ± 0.11 |  |  |  | -0.02 ± 0.12 |  | 207.63 | 5.14 | 0.05 | 0.02 |
| 57 | -0.1 ± 0.2 | 0.08 ± 0.18 | 0.32 ± 0.18 | -0.19 ± 0.12 | 0.2 ± 0.29 | 0.08 ± 0.13 |  | 207.79 | 5.3 | 0.10 | 0.02 |
| 6 | 0 ± 0.12 |  |  |  |  |  | 0.2 ± 0.12 | 208.29 | 5.8 | 0.00 | 0.03 |
| 45 | -0.11 ± 0.19 | 0.02 ± 0.18 | 0.32 ± 0.19 |  | 0.26 ± 0.14 | 0.08 ± 0.19 |  | 208.34 | 5.85 | 0.09 | 0.02 |
| 48 | -0.06 ± 0.18 | 0.31 ± 0.12 |  | -0.19 ± 0.29 | 0.07 ± 0.13 | 0 ± 0.18 |  | 208.91 | 6.42 | 0.07 | 0.02 |
| 29 | -0.06 ± 0.18 | 0.26 ± 0.12 |  |  | 0.13 ± 0.18 | 0 ± 0.12 |  | 209.42 | 6.93 | 0.06 | 0.02 |
| 18 | 0 ± 0.12 |  |  | -0.08 ± 0.12 |  |  | 0.2 ± 0.12 | 209.78 | 7.29 | 0.00 | 0.02 |
| 3 | -0.03 ± 0.17 |  |  | -0.1 ± 0.12 |  |  |  | 209.84 | 7.35 | 0.06 | 0.03 |
| 20 | -0.02 ± 0.14 |  |  |  | 0.08 ± 0.26 |  | 0.21 ± 0.14 | 210.19 | 7.7 | 0.00 | 0.03 |
| 21 | 0 ± 0.12 |  |  |  |  | -0.02 ± 0.12 | 0.2 ± 0.12 | 210.27 | 7.78 | 0.00 | 0.03 |
| 5 | -0.03 ± 0.16 |  |  |  |  | -0.03 ± 0.12 |  | 210.5 | 8.01 | 0.06 | 0.03 |
| 4 | -0.04 ± 0.18 |  |  |  | 0.06 ± 0.18 |  |  | 210.52 | 8.03 | 0.06 | 0.03 |
| 39 | -0.01 ± 0.14 |  |  | -0.08 ± 0.12 | 0.05 ± 0.14 |  | 0.2 ± 0.27 | 211.74 | 9.25 | 0.00 | 0.02 |
| 40 | 0 ± 0.12 |  |  | -0.08 ± 0.12 |  | -0.01 ± 0.12 | 0.2 ± 0.12 | 211.77 | 9.28 | 0.00 | 0.02 |
| 17 | -0.04 ± 0.17 |  |  | -0.1 ± 0.17 |  | -0.03 ± 0.12 |  | 211.79 | 9.3 | 0.06 | 0.03 |
| 16 | -0.04 ± 0.18 |  |  | -0.1 ± 0.18 | 0.02 ± 0.12 |  |  | 211.83 | 9.34 | 0.06 | 0.03 |
| 41 | -0.02 ± 0.14 |  |  |  | 0.08 ± 0.14 | 0 ± 0.29 | 0.21 ± 0.13 | 212.19 | 9.7 | 0.00 | 0.03 |
| 19 | -0.04 ± 0.18 |  |  |  | 0.04 ± 0.29 | -0.02 ± 0.13 |  | 212.49 | 10 | 0.06 | 0.03 |
| 56 | -0.01 ± 0.15 |  |  | -0.08 ± 0.13 | 0.05 ± 0.12 | 0 ± 0.15 | 0.2 ± 0.12 | 213.74 | 11.25 | 0.00 | 0.02 |
| 38 | -0.04 ± 0.19 |  |  | -0.1 ± 0.13 | 0 ± 0.19 | -0.03 ± 0.12 |  | 213.79 | 11.3 | 0.06 | 0.03 |
